# A Two-Stage Feature Point Detection and Marking Approach Based on the Labeled Multi-Bernoulli Filter

**DOI:** 10.3390/s22145083

**Published:** 2022-07-06

**Authors:** Jiahui Yang, Weifeng Liu

**Affiliations:** School of Electrical and Control Engineering, Shaanxi University of Science and Technology, Xi’an 710021, China; 200612041@sust.edu.cn

**Keywords:** LMB filter, L-RFS, feature point detection and marking

## Abstract

In recent years, various algorithms using random finite sets (RFS) to solve the issue of simultaneous localization and mapping (SLAM) have been proposed. Compared with the traditional method, the advantage of the RFS method is that it can avoid data association, landmark appearance and disappearance, missed detections, and false alarms in Bayesian recursion. There are many problems in the existing robot SLAM methods, such as low estimation accuracy, poor back-end optimization, etc. On the basis of previous studies, this paper presents a labeled random finite set (L-RFS) SLAM method. We describe a scene where the sensor moves along a given path and avoids obstacles based on the L-RFS framework. Then, we use the labeled multi-Bernoulli filter (LMB) to estimate the state of the sensor and feature points. At the same time, the B-spline curve is used to smooth the obstacle avoidance path of the sensor. The effectiveness of the algorithm is verified in the final simulation.

## 1. Introduction

Simultaneous localization and mapping (SLAM) is a process of gradually constructing an overall environmental map (feature point location, number, etc.) based on the detection data of the mobile sensor. Feature points are targets that need to be detected by the sensor in a given scene. The sensor also needs to estimate its location simultaneously. Nowadays, SLAM plays an extremely important role in many fields. It is considered to be a major process required by many mobile robot systems. Perception, positioning, and mapping are the key problems in unmanned driving [1], autonomous navigation [2], mining [3], agriculture [4], and many other fields. In the SLAM process, positioning is the estimation of the sensor position; the primary premise of mapping is to estimate the state of feature points in the given scene.

The SLAM issue was first put forward by Hugh Durrant-Whyte and John J. Leonard in 1986. The development of SLAM technology has gone through the early era (1986–2004), the algorithm analysis era (2004–2015), and the robustness era (2015-). There has been some review literature about SLAM frameworks and methods, including more classic ones such as articles written by Cadena et al. [5]. The SLAM problem can be described as follows: A robot moves in an unknown environment. Its position will be determined through the observation of the surrounding environment, and an environmental map will be constructed at the same time. In general, a SLAM system includes the following three processes: perception, positioning, and mapping. Perception is a prerequisite. It is mainly used to determine positioning and navigation in an unknown environment. Robot positioning and map construction are two mutually dependent and complementary processes.

In the field of autonomous navigation and unmanned driving, it is very important to estimate the location of feature points in the surrounding environment. The main theories are based on the Bayes theory. They can be classified into three classes: the traditional Bayes methods, the Bayesian nonparametric estimation methods, and the random finite sets (RFS)-based methods. 

In traditional Bayesian estimation problems, there are two types of filtering algorithms commonly used. One is suitable for nonlinear problems. Various parametric filtering algorithms were developed on the basis of the Kalman filtering algorithm, such as extended Kalman filtering, Linear Kalman filtering, etc.; the other is a nonparametric filtering algorithm based on Monte Carlo simulation technology, such as particle filtering. Drovandi, C. et al. [6] presented a sequential Monte Carlo (SMC) algorithm that can be used for anyone-at-a-time Bayesian sequential design problem in the presence of model uncertainty where discrete data are encountered. A SMC algorithm is run in parallel for each model and the algorithm relies on a convenient estimator of the evidence of each model, which is essentially a function of importance sampling weights. Martino, L. et al. [7] designed a sequential Monte Carlo scheme for the dual purpose of Bayesian inference and model selection. They use interacting parallel particle filters, with each one addressing a different model. Urteaga, I. et al. [8] proposed a Sequential Monte Carlo (SMC) method for the filtering and prediction of time-varying signals under model uncertainty. Instead of resorting to model selection, they fuse the information from the considered models within the proposed SMC method. The particle filter algorithm will theoretically give better estimation results, but at an expensive computational cost. This method also has many drawbacks: it relies heavily on the estimation of the initial state and may converge or diverge very quickly. In addition, there is a particle degradation problem. Due to weight sampling, there are problems such as the lack of particles and loss of some data.

In recent years, many Bayesian nonparametric estimation methods have emerged to solve the MOT problem. Moraffah, B. et al. [9] proposed a new method in which they use information from previously tracked objects to track the dynamically changing number of objects. The method is based on nonparametric Bayesian modeling using diffusion processes and random trees. In the same year, Moraffah, B. also proposed robust nonparametric methods to model state priors for MOT problems [10]. These models are shown to be more flexible and robust than existing methods. In particular, the holistic approach estimates time-dependent object cardinality, provides object labels, and identifies object-dependent measurements. Furthermore, their framework dynamically competes with object birth/death and survival through correlated nonparametric processes. Yi, S. et al. [11] considered the robust state space filtering problem in the case where the transition probability density is unknown and possibly degenerate. The resulting robust filter has a Kalman-like structure and solves a minimax game; the least popular model is then naturally selected in the prescribed ambiguity set, which also contains non-Gaussian probability densities, while the other participants design the best filters for the least popular models. Hjalmarsson, H. et al. [12] suggested a simple and explicit method for estimating the model uncertainty, which is also applicable to severe under-modelling. A generalized Kalman-Bucy model under model uncertainty and a corresponding robust problem are studied in the literature [13]. Ji, S. et al. found that this robust problem is equivalent to an estimate problem under a sublinear operator. By using a Girsanov transformation and the minimax theorem, they prove that this problem can be reformulated as a classical Kalman-Bucy filtering problem under a new probability measure.

In recent years, many researchers have introduced the RFS formula in SLAM systems. Kalyan [14] described SLAM topics by using the Finite Set Statistics (FISST) framework. He used RFS to describe the state of feature points. Furthermore, the problem of mutual fusion between local maps can also be represented by RFS. Leung, K. [15] proposed RFS to represent the problem of feature point state estimation in positioning, mapping, and tracking. Under poor detection conditions, he provided a more robust solution. Inostroza, F. [16] proposed RFS to solve the data association problem in maximum likelihood SLAM. At the same time, he also proved to readers that maximum likelihood SLAM can be used without the need for external data association algorithms. Hampton, B. et al. [17] described the implementation of a tiny open-source and cost-effective SLAM robot. The robot utilized a novel occupancy grid SLAM algorithm based on the concept of RFS. Moratuwage, D. et al. [18] extended the SLAM filter framework based on RFS using a multi-sensor information fusion method and proposed a new solution to the multi-vehicle SLAM (MVSLAM) problem. They modelled measurements and landmark maps as RFS and factorized the MVSLAM posterior into the product of the joint vehicle trajectory posterior and the landmark map posterior conditioned on the vehicle trajectory. Du Hang yuan [19] described the status information, observation information, and environmental map in SLAM as RFS form. Subsequently, he proposed probability hypothesis density (PHD) to estimate the state of feature points under the framework of Bayes estimation. When the number of feature points in the environment is unknown, Mullane [20] proposed the SLAM Bayesian framework. The key to this framework modeling is to represent the map as a set of limited feature values. Using RFS theory, the SLAM problem will be transformed into a posterior estimation problem based on Bayesian filtering.

Starting from the point process theory, Mahler [21] proposed the theoretical method tool of Finite Set Statistics (FISST), which provided a theoretical basis for engineering calculations for target tracking and detection using RFS. Due to the low accuracy of robot mapping in environments with dense clutter and many map feature points, the literature [22] proposed a RFS SLAM method based on amplitude information. This method used the amplitude information measured by the map feature to obtain the likelihood function of the map feature and clutter. It is used to improve the estimation accuracy of the feature map in the SLAM process. In the PHD prediction stage, the map that has been observed before the last moment is used as the prior information of the prediction stage. Adding the prior information improves the estimation accuracy of the position and number of feature points [23]. In Bayesian filtering, Li, T. et al. [24] considered the rebirth, death, and regeneration of feature points, and gave the birth intensity of feature points to improve the detection accuracy of the sensor.

FISST has laid a scientific and rigorous theoretical foundation for the multi-target tracking problem. Among them, based on the RFS and the probability statistics of sets, the multi-target Bayesian equation for the multi-target tracking problem can be obtained [21,25]. In the multi-target tracking process, the number of targets may change with time and the state of each target is also constantly changing. The number of measurements also changes randomly and each measurement itself can be regarded as a random variable. The statistical model of the problem can be constructed as RFS, and the complex data association process can be effectively solved. In multitarget tracking, a closed form solution to the Bayes multitarget filter, which can also output target tracks, is given in [26,27,28]. The main breakthrough is the first multitarget conjugate prior with respect to the standard multitarget likelihood function. The prior is called the generalized labeled multi-Bernoulli prior. Additionally, this multi-target prior is also closed under the Chapman-Kolmogorov equation for the standard multi-target transition density [27].

In multi-target tracking and labeling, the objective is to jointly estimate the number of trajectories and their states from a sequence of noisy and cluttered observation sets. A multi-target system is fundamentally different from a single-target system in that the number of states changes with time due to births and deaths of targets. In addition, existing targets may or may not be detected and the sensor also receives a set of spurious measurements (clutter) not originating from any target. As a result, at each time step the measurement is a collection of detections, only some of which are generated by targets. Stephan R et al. [29] proposed a generalization of the multi-Bernoulli filter called the labeled multi-Bernoulli filter that outputs target tracks. Moreover, the labeled multi-Bernoulli filter does not exhibit a cardinality bias due to a more accurate update approximation compared to the multi-Bernoulli filter by exploiting the conjugate prior form for labeled Random Finite Sets. The proposed filter can be interpreted as an efficient approximation of the δ-Generalized Labeled Multi-Bernoulli filter. It inherits the advantages of the multi-Bernoulli filter in regard to particle implementation and state estimation. It also inherits the advantages of the δ-Generalized Labeled Multi-Bernoulli filter in that it outputs (labeled) target tracks and achieves better performance. When it comes to the application of the LMB filtering method to the SLAM problems, the LMB filter was introduced as an efficient approximation of the computationally expensive δ-Generalized LMB (δ-GLMB) filter. The LMB filter converts its representation of an LMB distribution to the δ-GLMB form and back during the measurement update step. Hendrik, D. et al. [30] addressed the simultaneous localization and mapping (SLAM) problem and proposed a Rao-Blackwellized implementation of the Labeled Multi-Bernoulli SLAM (LMB-SLAM) filter. Further, they established that the LMB-SLAM does not require the approximations used in Probability Hypothesis Density SLAM (PHD-SLAM). Moratuwage, D. et al. [31] presented a SLAM solution using an efficient variant of the δ-GLMB filter (δ-GLMB-SLAM) based on Gibbs sampling, which is computationally comparable to LMB-SLAM, yet more accurate and robust against sensor noise, measurement clutter, and feature detection uncertainty. Herrmann, M. et al. [32] described a novel method to additionally incorporate multiple hypotheses for fusing the measurements of the object reference points using an extension to the previously presented Labeled Multi-Bernoulli (LMB) filter.

By treating the map as an RFS and updating the first-order moment of its multi-target density, a local SLAM method based on random sets is used on each sensor. The map is regarded as a random finite set and the first-order moment of its multi-object density is updated, which is called PHD. The consensus on the map PHDs is adopted to disseminate map information through the sensor team, while also taking into account the different and time-varying fields of view of the team members [33]. Since low-cost sensors are prone to missed detections and false alarms, Ristic B. et al. [34] proposed an occupied grid algorithm for SLAM systems. The algorithm used the RFS framework to describe sensor observation information. The Rao-Blackwellised particle filter is also used to estimate the sensor state. Based on the existing theory, this paper proposes a two-stage SLAM feature point detection and marking algorithm based on LMB filtering. The first stage is to plan the sensor’s initial path according to the given scene size. We refer to the feature points that appear on the initial path as obstacles. In the second stage of path planning, the sensor avoids obstacles during movement and detects the position of obstacles. Subsequently, LMB filtering is used to estimate the state of sensors and feature points. The B-spline curve is used to smooth the path of obstacle avoidance. Finally, the algorithm was verified.

The main innovation of our paper is to provide a two-stage algorithm based on the labeled RFS theory for the SLAM problem; specifically, we divide the sensor path planning into rough and refined planning stages. Considering the limited detection field for the mobile sensor platform, we use the LMB filtering method to estimate the state of the feature points for its simplicity and relatively better precision.

The structure of this paper is organized as follows: We explain relevant background knowledge and problem descriptions, including L-RFS and SLAM problem descriptions, in Section 2. Subsequently, in Section 3, we describe the two-stage sensor path planning (initial path planning and local obstacle avoidance) and LMB filtering algorithm in detail. Section 4 describes the algorithm simulation and the simulation results are organized into charts to illustrate the effectiveness of the algorithm in this paper. Section 5 is the conclusion of this paper.

## 2. Background Knowledge and Problem Description

### 2.1. L-RFS Model for Map Observation

The robot positioning process in the SLAM system is to estimate the position of the robot when the external environment map is known. On the contrary, in the process of mapping, the state of the robot or the sensor is known. During the movement of the sensor, the measurement information will be acquired and saved, and will be used to estimate the state of the feature points. Accurate positioning requires an unbiased map, but such a map also requires accurate location estimation to describe. The SLAM system combines the two processes of positioning and mapping, so the states of robots or sensors and feature points must be estimated together. These two processes are complementary and inseparable.

In the RFS framework, the number of elements in an RFS variable is random, and the elements themselves are random and disordered. $X_{k}$ and $Z_{k}$ are defined as a finite set composed of state information and observation information. In the case of extending from a single feature point to multiple feature points, the feature point status information can be described as a set [35,36]:

(1) $$X_{k}=\{x_{k,1},x_{k,2},\ldots ,x_{k,i}\}\in F(X)$$

$x_{k,i}$ represents the state of the i-th feature point at time k. The state set $X_{k}$ contains state information of multiple feature points at time k. The RFS of the feature point state can be described by the following model:

(2) $$X_{k}=[\cup_{x\in X_{k-1}}S_{k|k-1}(x)]\cup [\cup_{x\in X_{k-1}}B_{k|k-1}(x)]\cup \Gamma_{k}(x)$$

where $S_{k}(x)$ represents the survival feature points, $B_{k}(x)$ represents the newly born feature points, and $\Gamma_{k}\left(x\right)$ represents the regenerated feature points at time.

Similarly, the measurement RFS can be modeled as:

(3) $$Z_{k}=\{z_{k,1},z_{k,2},\ldots ,z_{k,j}\}\in F(Z)$$

$z_{k,i}$ represents the measured value of the i-th feature point at time k. i and j are the true value and the measured value of the number of feature points, respectively. Due to missed detections and false alarms, i and j are not necessarily the same. F(X) and F(Z) are the collections of the state space X and the finite subset of the observation space Z, respectively.

Unlike traditional RFS, L-RFS adds a unique label $l\in L=\{\alpha_{i},i\in N\}$ to the state space. The following formula represents the static characteristic RFS of the entire environment map,

(4) $$M_{k}=\{m_{k,1}^{},m_{k,2}^{},\ldots ,m_{k,i}^{}\}$$

where $m_{k,1}^{},m_{k,2}^{},\ldots ,m_{k,n_{m}}^{}$ is the feature vector at time k. $M_{k-1}$ is a subset of the map that has been traversed, and the currently observed features are represented by $C\left(x_{k}\right)$.

(5) $$M_{k}=M_{k-1}\cap C(x_{0:k-1})\;$$

where $C(x_{0:k-1})=C(x_{0})\cup C(x_{1})\cup \ldots \cup C(x_{k-1})$. Therefore, $M_{k-1}$ represents the union of each observation feature space before k−1, so the traversal process map or feature update equation is:

(6) $$M_{k}=M_{k-1}\cup (C(x_{k-1})\cap {\bar{M}}_{k-1})$$

where ${\bar{M}}_{k-1}=M-M_{k-1}$. $C(x_{k-1})\cap {\bar{M}}_{k-1}$ is the newly observed feature point, which can be expressed by independent RFS, and the transfer density of the RFS map can be expressed as:

(7) $$f_{M}(M_{k}|M_{k-1},x_{k})=\sum_{w\subseteq M_{k}}f_{M}(w|M_{k-1})f_{B}(M_{k}-w|x_{k})$$

where $f_{M}(w|M_{k-1})$ is the transition density of the feature set in the detection range from time k−1 to time k; $f_{B}(M_{k}-w|x_{k})$ is the density of the new feature $B_{k}(x_{k})$ in the detection range at time.

### 2.2. Mobile Robot SLAM Problem Description

**Figure 1:**
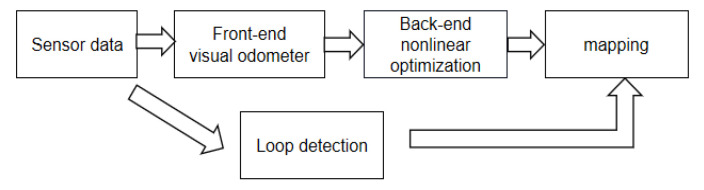
The SLAM Classic Framework. (The framework is mainly composed of the following five parts: sensor, visual odometer, back-end, closed-loop detection, and mapping. Their respective roles are explained in detail in the paragraphs after Figure 1).

SLAM technology based on Lidar and visual images has become an indispensable new technology in current SLAM systems. Because it has many advantages, visual SLAM can accurately obtain the state of feature points, without prearranging the scene, and can fuse multiple sensors. Laser SLAM can work in poor light environments and generate an occupation grid map that is easy to navigate [37]. There are two main tasks of laser SLAM: one is to estimate the position of the subject carrying the lidar in motion, and the other is to simultaneously build a map of the surrounding environment. Accurate positioning requires an accurate map. An accurate map comes from accurate positioning. Positioning focuses on self-understanding, while mapping focuses on external understanding. The classic framework of SLAM is mainly composed of the following five parts: sensor, visual odometer, back-end, closed-loop detection, and mapping, as shown in the Figure 1 [38].

Sensors and visual odometers are called front-end in visual SLAM. Sensors are mainly used to collect environmental information. The purpose of the visual odometer is to estimate its state based on the image data collected by the sensor. The formed pose information at different moments is sorted and optimized to obtain a complete environmental map. The loop detection link is obviously a feedback link. It compares the information of the sensor, the visual odometer, and the back-end to determine whether there is drift or repetition in the position of the robot’s movement. If there is, it will feedback its information to the back-end to reduce the cumulative error [38].

In the SLAM system, we can use the Bayesian framework to describe and define two variables for the sensor: one is the state quantity, the other is the observation measurement. State variables: $x_{1:k}≅\left(x_{1},x_{2}\ldots \ldots x_{k}\right)$, observed variables: $z_{1:k}≅\left(z_{1},z_{2}\ldots \ldots z_{k}\right)$. Among them, the state quantity refers to the posture, position velocity, and three-dimensional point coordinates of the sensor at each moment, and $x_{k}$ refers specifically to the state at time k. The observation is the position of the feature point detected by the sensor in the current state. Similarly, $z_{k}$ is the observation at time k.

SLAM can be modeled as the state estimation problem of the robot system:

(8) $$x_{k}=f(x_{k-1},u_{k},w_{k})\;k=1,\ldots ,K\;z_{k,j}=h(m_{j},x_{k},v_{k,j})\;k=0,\ldots ,K$$

where $x_{k}$ is the pose of the robot at time k; $u_{k}$ is the control input; $w_{k}$ is the measurement noise; $z_{k,j}$ is the observation of the environmental feature point $m_{j}$ at time k; j is the number of feature points; and $v_{k,j}$ is the observation noise. Using the control amount $u_{k}$ of the driver and the environmental observation data $z_{k,j}$ eliminates the influence of noises such as $w_{k}$ and $v_{k,j}$. The state of the robot system, namely the pose $x_{k}$ and the feature point $m_{j}$, is estimated for the uncertainty of the state space, thereby obtaining the estimate of the $x_{k}$ sequence. This process is called positioning. The process of estimating the $m_{j}$ sequence is called mapping.

## 3. Two-Stage Feature Point Detection and Estimation Algorithm Based on the LMB Filter

### 3.1. Two-Stage Path Planning for Sensor

The algorithm proposed in this paper is the two-stage path planning based on event triggering. The sensor is divided into two modes: driving mode and obstacle avoidance mode. The first stage is the initial path planning, focusing on the overall situation. At this time, the sensor enters the driving mode and drives normally according to the given route. For initial path planning, all information about the scene is known in advance and this information is used as the basis for path planning. In other words, an optimal path is found between the starting point and the ending point according to the research purpose. Thus, in the process of driving, the detection range of the sensor covers the entire scene to the maximum extent. The accuracy of path planning depends on the accuracy of environmental information acquisition. Although the planning result is overall better, it is not detailed enough. If the sensor detects an obstacle during the movement, it triggers the second phase of local path planning. The sensor enters obstacle avoidance mode. In this process, the sensor uses the measurement information to update and adjust the original path locally based on the initial path, avoiding obstacles in real time. After successfully avoiding the obstacle, the sensor returns to the driving mode and continues driving according to the initial path. There is no essential difference between initial path planning and partial path planning. The sensor works in stages and can better plan the walking path from the start point to the end point.

### 3.2. Mobile Sensor Modeling

According to the finite coverage theorem, it is known that if H is an (infinite) open coverage of closed interval $\left[a,b\right]$, then a finite number of open intervals can be selected from H to cover interval $\left[a,b\right]$. The limited coverage theorem is a useful and important theorem. It is an important method to deal with mathematical analysis problems, and it has a wide range of applications in various fields of mathematics. The function of the finite coverage theorem is to select a finite number of open intervals from an infinite number of open intervals covering a closed interval, and also cover this closed interval [39].

In this paper, the sensor is modeled as a constant-speed straight line model (CV) and a constant-speed turning model (CT). The sensor follows the following dynamic model during driving:

(9) $$\left\{\begin{array}{l} x_{k+1}=F_{k}x_{k}+G_{k}u_{k}+v_{k}\\ u_{k+1}=L_{k}u_{k}\\ y_{k+1}=O_{k+1}x_{k+1}+w_{y,k+1} \end{array}\right.$$

where $F_{k}$, $G_{k}$, $L_{k}$, and $O_{k}$ are coefficient matrices. $v_{k}$ and $w_{y,k+1}$ are system noises. $u_{k}$ is the control input at time k. $x_{k}$ is the sensor state at time k. $y_{k+1}$ is the observation of the sensor at time k+1. When the sensor moves in a straight line,

$$F_{k}=\left[\begin{array}{cc} 1 & 0\\ 0 & 1 \end{array}\right],\;G_{k}=\left[\begin{array}{cc} T & 0\\ 0 & T \end{array}\right],\;L_{k}=\left[\begin{array}{cc} 1 & 0\\ 0 & 1 \end{array}\right],\;O_{k+1}=\left[\begin{array}{cc} 1 & 0\\ 0 & 1 \end{array}\right]$$

When the sensor moves along a circular arc with a turning rate w,

$$F_{k}=\left[\begin{array}{cc} 1 & 0\\ 0 & 1 \end{array}\right],\;L_{k}=\left[\begin{array}{cc} coswT & -sinwT\\ \mathrm{sinwT} & coswT \end{array}\right],\;\;G_{k}=\left[\begin{array}{cc} \frac{sinwT}{w} & -\frac{1-coswT}{w}\\ \frac{1-coswT}{w} & \frac{sinwT}{w} \end{array}\right],\;O_{k+1}=\left[\begin{array}{cc} 1 & 0\\ 0 & 1 \end{array}\right]$$

where T is the period and w is the angular velocity of the sensor movement.

The observation model is:

(10) $$z_{k+1}=\left\{\begin{array}{l} Hm_{k}+w_{k},\;𝓅(m_{k})\in S\overset{\Delta }{=}R(𝓅(x_{k}),r_{k})\\ \emptyset ,\;𝓅(m_{k})\notin S\overset{\Delta }{=}R(𝓅(x_{k}),r_{k}) \end{array}\right.$$

$$H=\left[\begin{array}{cccc} 1 & 0 & 0 & 0\\ 0 & 0 & 1 & 0 \end{array}\right]$$

where 𝓅(⋅) is the position function and S represents the detection area of the sensor. $R(𝓅(x_{k}),r_{k})$ represents a circular area where the obstacle position is the center of the circle and $r_{k}$ is the radius. $w_{k}$ is noise.

### 3.3. Feature Points Estimation Method Based on LMB

Both unlabeled RFS and labeled RFS belong to the random finite point process [29,30]. The LMB filter can realize the requirement of feature point detection and marking.

First, add the label in the feature point state set, $M=\{(m_{i},l_{i})\}$ $(i=1,2,\ldots ,m),m_{i}\in M,\;l_{i}\in L$, M is the feature point state space, L is the label space, and |⋅| represents the potential of the set. LMB RFS can be represented by parameter $\left\{(r^{\left(l\right)},p^{\left(l\right)}(\cdot ),l\in L\right\}$, where $r^{\left(l\right)}\in [0,1]$ is the probability of the l-th feature point label, assuming that the position is the true position. The LMB RFS density is as follows [40,41,42]:

(11) $$\pi (Χ)=\Delta (Χ)w(L(X))p^{\left\{Χ\right\}}$$

(12) $$\Delta (\left\{Χ\right\})=\delta_{\left|\left\{Χ\right\}\right|}(|L(\left\{Χ\right\})|)$$

(13) $$w(L)=\prod_{i\in L}\left(1-r^{i}\right)\prod_{l\in L}\frac{1_{L}(l)r^{l}}{1-r^{l}}$$

(14) $$p^{Χ}\overset{\Delta }{=}\prod_{(x,l)\in Χ}p^{\left(l\right)}(x)$$

where Δ(M) is a discrete label indicator; L(M) is a label set; w(L(M)) is a weighting coefficient and depends on the status label L(M); and $1_{L}(M)$ is an indicator function, when M⊆L is equal to 1, and others are equal to 0.

The LMB filter algorithm is used to detect and estimate the feature points. The LMB filter is mainly divided into two steps: the prediction step and the update step [43,44]. The feature point state is (m,l), the probability of a feature point being detected is $p_{D}(m,l)$, and the probability of not being detected is $q_{D}(m,l)=1-p_{D}(m,l)$.

**Prediction:** Consider the survival and rebirth of feature points here, so the parameters of LMB predicting the density of multiple feature points can be expressed as:

(15) $$\pi_{+}={\left\{(r_{+,S}^{\left(l\right)},p_{+,S}^{\left(l\right)})\right\}}_{l\in L}\cup {\left\{(r_{B}^{\left(l\right)},p_{B}^{\left(l\right)})\right\}}_{l\in B}$$

(16) $$r_{+,S}^{\left(l\right)}=\eta_{S}(l)r^{\left(l\right)}$$

(17) $$p_{+,S}^{\left(l\right)}=\langle p_{S}(\cdot ,l),f(m|\cdot ,l)\rangle /\eta_{S}(l)$$

(18) $$\eta_{S}(l)=\langle p_{S}(\cdot ,l),p^{\left(l\right)}(\cdot )\rangle$$

The LMB parameter for predicting feature points is $\left\{r_{+}^{\left(l\right)},{\left\{w_{+,j}^{\left(l\right)},m_{+,j}^{\left(l\right)}\right\}}_{j=1}^{J_{+}^{\left(l\right)}}\right\}$, where $L_{+}=L\cup B$; $\langle \cdot ,\cdot \rangle$ is the inner product function.

Update: Suppose that the multi-feature point prediction is recorded as the LMB RFS defined in the state space $M_{+}$ and the label space $L_{+}$, the parameter set is then expressed as follows:

(19) $$\pi_{+}={\left\{(r_{+}^{\left(l\right)},p_{+}^{\left(l\right)})\right\}}_{l\in L_{+}}$$

The posterior density of multiple feature points can be expressed as [40]:

(20) $$\pi (\cdot |Z)={\left\{(r^{\left(l\right)},p^{\left(l\right)}(m))\right\}}_{l\in L_{+}}$$

(21) $$r^{\left(l\right)}=\sum_{(I_{+},\theta )\in F(L_{+})\times \Theta_{I_{+}}}w^{\left(I_{+},\theta \right)}(Z)1_{I_{+}}(l)$$

(22) $$p^{\left(l\right)}(m)=\frac{1}{r^{\left(l\right)}}\times \sum_{(I_{+},\theta )\in F(L_{+})\times \Theta_{I_{+}}}w^{\left(I_{+},\theta \right)}(Z)\times 1_{I_{+}}(l)p^{\left(\theta \right)}(m,l|Z)$$

(23) $$w^{\left(I_{+},\theta \right)}(Z)\propto w_{+}(I_{+}){\left[\eta_{Z}^{\left(\theta \right)}\right]}^{I_{+}}$$

(24) $$p^{\left(\theta \right)}(x,l|Z)=\frac{p_{+}(m,l)\psi_{Z}(m,l;\theta )}{\eta_{Z}^{\left(\theta \right)}(l)}$$

(25) $$\eta_{Z}^{\left(\theta \right)}(l)=\langle p_{+}(m,l)\psi_{Z}(m,l;\theta )\rangle$$

(26) $$\psi_{Z}(m,l;\theta )=\left\{\begin{array}{l} \frac{p_{D}(m,l)g(z_{\theta (l)}|m,l)}{K(z_{\theta (l)})},\theta (l)>0\\ q_{D}(m,l),\theta (l)=0 \end{array}\right.$$

where $p_{D}(m,l)$ is the probability that the feature point labeled l is detected, and $q_{D}(m,l)=1-p_{D}(m,l)$ is the probability that the feature point m labeled l is not detected. g(z|m,l) is the single feature point likelihood function of the feature point m labeled l with respect to the measurement z. θ represents the mapping from the label set to the measurement set. K(⋅) represents the clutter function.

### 3.4. Obstacle Avoidance Strategy Based on Event Triggering

In the two-stage path planning mentioned in this article, the sensor is switched between the driving mode and the obstacle avoidance mode by event triggering, and the sensor enters the driving mode in the first stage and travels according to the given path. When formula (29) is satisfied, the sensor switches to the second stage obstacle avoidance mode.

(27) $$||𝓅(x_{k})-𝓅(m_{k})|{|}_{2}\leq \sigma_{min}$$

(28) $$K=min\{k:||𝓅(x_{k})-𝓅(m_{k})|{|}_{2}\leq \sigma_{min}\}$$

(29) $$𝓅(y_{k})=𝓅(x_{k})+\sigma_{min}$$

where $\sigma_{min}$ is the safety distance, which is a constant. $||𝓅(x_{k})-𝓅(m_{k})|{|}_{2}$ indicates the distance between the feature point and the sensor. When formula (28) is satisfied, the sensor switches from the driving mode to the mode of obstacle avoidance. Here, time is denoted as k, and the minimum value is the time of event triggering, denoted as K. In the obstacle avoidance phase, the preview point $y_{k}$ is first given. When the sensor travels to the preview point, it exits the obstacle avoidance mode and enters the driving mode.

**Figure 2:**
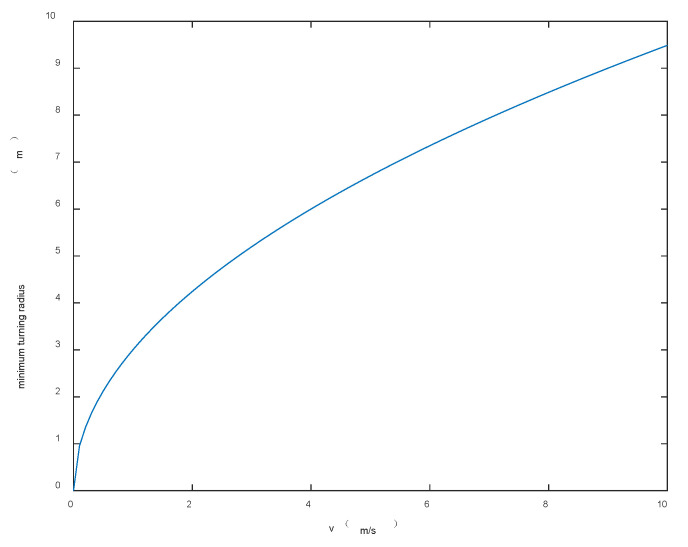
The relationship between sensor obstacle avoidance radius and speed.

Assuming that the radius of all obstacles in the scene is not greater than 5 m, the following figure shows the relationship curve between sensor obstacle avoidance radius r and driving speed v. As shown in Figure 2, the two are directly proportional, that is, the faster the driving speed, the larger the obstacle avoidance radius, and correspondingly, the larger $\sigma_{min}$.

After determining the obstacle avoidance radius, the initial straight path is directly connected with the sensor obstacle avoidance arc path. This will cause the sensor to instantly change 90° in the direction of driving at the junction of the two paths. Sensors on the move cannot do this. Therefore, this paper uses the B-spline curve fitting method to smooth the path. The B-spline curve is widely used in path planning and it is an effective path optimization method. It has the characteristics of geometric invariance, convexity, and reduced deterioration. The B-spline curve is described by the following formula [41]:

(30) $$C(u)=\sum_{i=0}^{n}N_{i,k}(u)X(i)$$

(31) $$N_{i,k}(u)=\left\{\begin{array}{l} 1,u_{i}\leq u\leq u_{i+1}\\ 0,otherwise \end{array}\right.$$

(32) $$N_{i,k}(u)=\frac{u-u_{i}}{u_{i+k}-u_{i}}N_{i,k-1}(u)+\frac{u_{i+k+1}-u}{u_{i+k+1}-u_{i+1}}N_{i,k+1}(u)$$

(33) $$define\frac{0}{0}=0$$

(34) $$u_{0}\leq u_{1}\leq \ldots \leq u_{m-1}\leq u_{m}$$

where $C\left(u\right)$ indicates the fitted curve. k is the order of the spline curve, $u_{i}$ is the node on the curve after fitting, X(i)) is the path point to be fitted, and $N_{i,k}(u)$ is the basis function.

## 4. Algorithm Simulation and Verification

**Figure 3:**
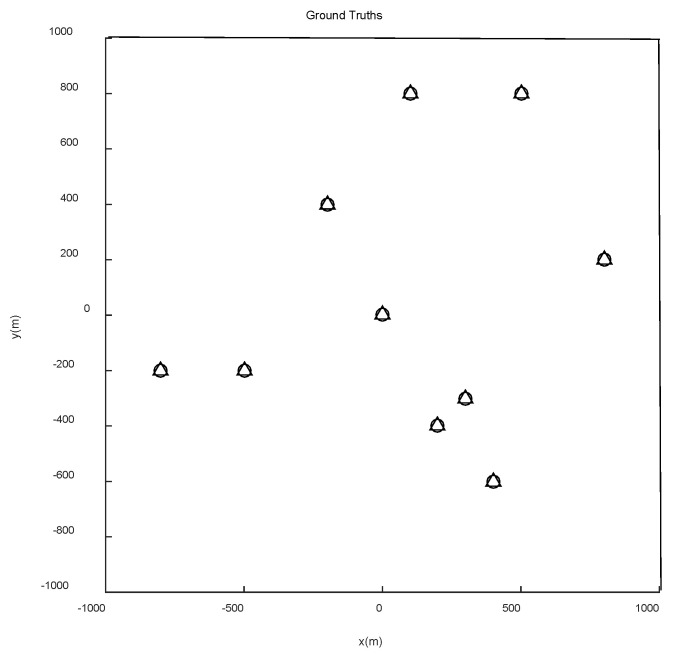
The true position of the feature points in the scene.

**Figure 4:**
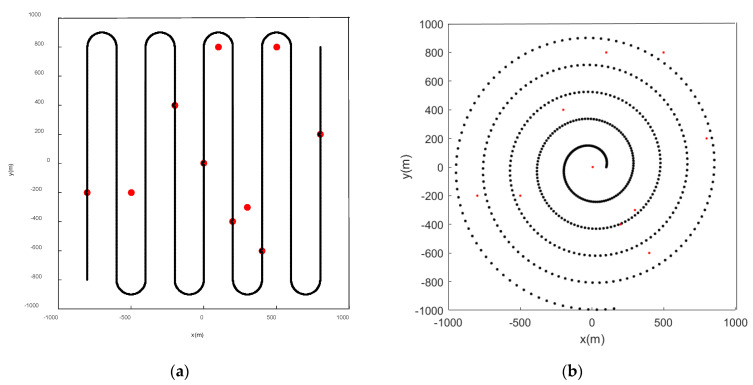
Initial path planning: (**a**) Serpentine curve; (**b**) Spiral curve.

**Figure 5:**
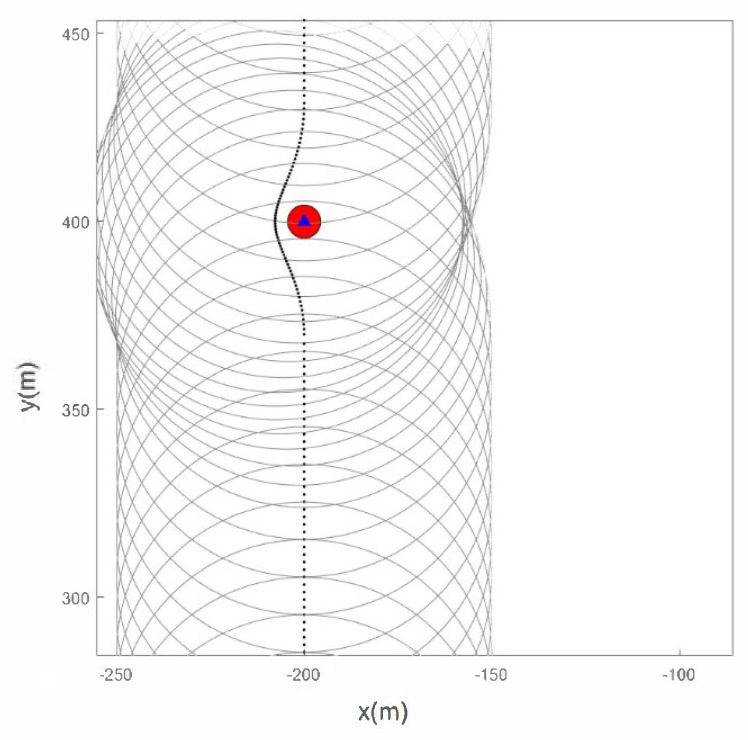
Sensor avoidance.

The scene given in this paper is a square area with multiple feature points. The locations of the feature points are shown in Figure 3. The algorithm detects and labels feature points based on some parameters such as size and shape of a given scene. The straight-line part is the CV model, and the arc part is the CT model. As shown in Figure 4, the initial position of the path is (−800 m, −800 m) and the end position is (800 m, 800 m). According to the above obstacle avoidance strategy, the schematic diagram of sensor obstacle avoidance is shown in Figure 5.

Set the sensor to detect along the initial path shown in Figure 4. The parameter settings are shown in the table below. 

All parameters in Table 1 can be adjusted at any time according to the number of obstacles in the scene and changes in position distribution.

We use the B-spline curve in the obstacle avoidance phase to smooth the sensor path. It can be observed from Figure 5 that according to the obstacle avoidance strategy mentioned above, the sensor effectively avoids the characteristic points appearing on the initial path.

**Figure 6:**
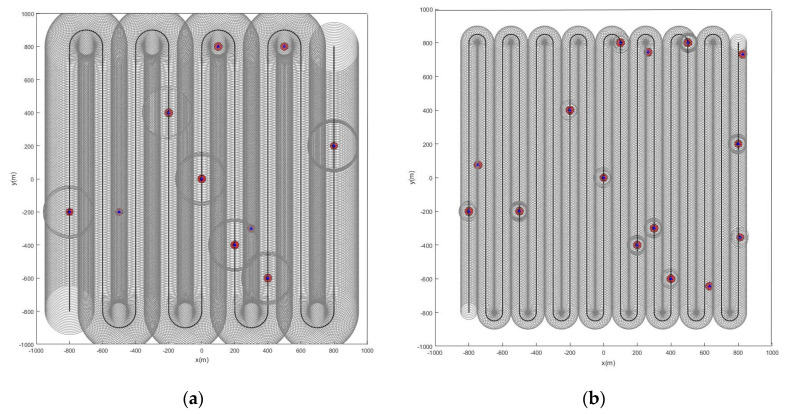
Estimated position of stationary feature points using the LMB algorithm (the red and blue marks indicate the true and estimated positions, respectively, of the feature points in the scene): (**a**) the detection radius of the sensor is 150 m, there are 10 feature points in the scene; (**b**) sensor detection radius is 50 m, there are 15 feature points in the scene.

**Figure 7:**
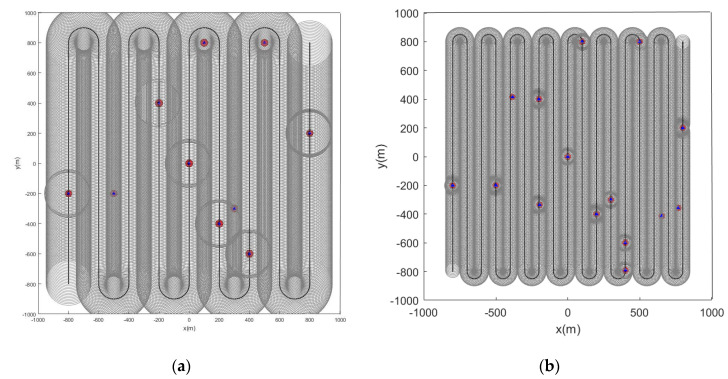
Estimated position of stationary feature points using the GLMB algorithm (the red and blue marks indicate the true and estimated positions, respectively, of the feature points in the scene): (**a**) the detection radius of the sensor is 150 m, there are 10 feature points in the scene; (**b**) sensor detection radius is 50 m, there are 15 feature points in the scene.

When the initial path is a serpentine curve,, based on the initial path, we will verify whether the algorithm proposed in this paper can accurately detect the marked feature points. When the feature points in the scene are static, Figure 6 and Figure 7 are the simulation results obtained by using the LMB filtering method and the GLMB filtering method respectively.

**Figure 8:**
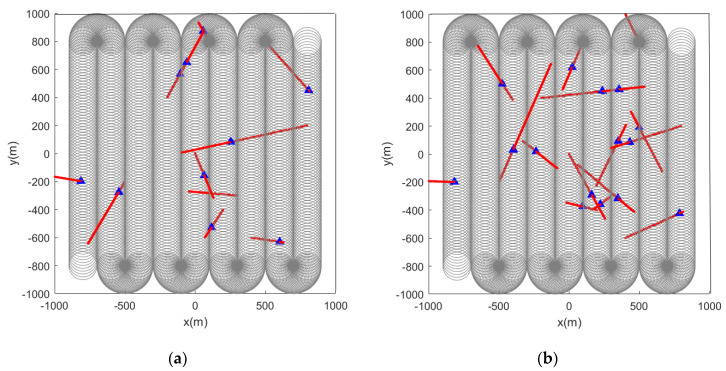
Estimated position of dynamic feature points using the LMB algorithm (the red track is the feature point motion track; the blue marks indicate the estimated positions of the feature points in the scene). (**a**) Sensor detection radius is 100 m, there are 10 feature points in the scene, and the red track is the feature point motion track; (**b**) sensor detection radius is 100 m, there are 15 feature points in the scene, and the red track is the feature point motion track.

**Figure 9:**
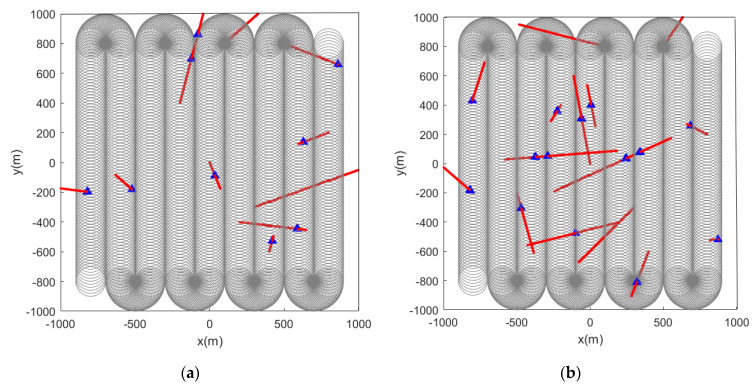
Estimated position of dynamic feature points using the GLMB algorithm (the red track is the feature point motion track; the blue marks indicate the estimated positions of the feature points in the scene). (**a**) Sensor detection radius is 100 m, there are 10 feature points in the scene, and the red track is the feature point motion track; (**b**) sensor detection radius is 100 m, there are 15 feature points in the scene, and the red track is the feature point motion track.

When the feature points are changed from static to dynamic, the simulation results are shown in Figure 8 and Figure 9.

Considering the scene where multiple feature points are moving, the number of feature points detected by the sensor changes all the time and the probability that the feature points are detected is $p_{D}=0.98$. The clutter is uniformly distributed, the clutter intensity is set to 20, the clutter area $S=\left[-1000,1000]\times [-1000,1000\right]$, and the process noise $Q_{k}=0.5^{2}\times B\times B^{T}$. The dynamic feature points make uniform linear motion in the two-dimensional plane, and the detection time is 100 s.

**Figure 10:**
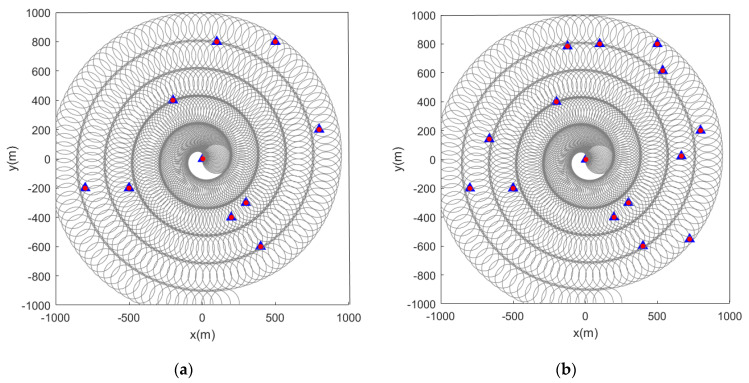
Estimated position of stationary feature points using the LMB algorithm (the red and blue marks indicate the true and estimated positions, respectively, of the feature points in the scene). (**a**) Sensor detection radius is 100 m and there are 10 feature points in the scene; (**b**) sensor detection radius is 100 m and there are 15 feature points in the scene.

**Figure 11:**
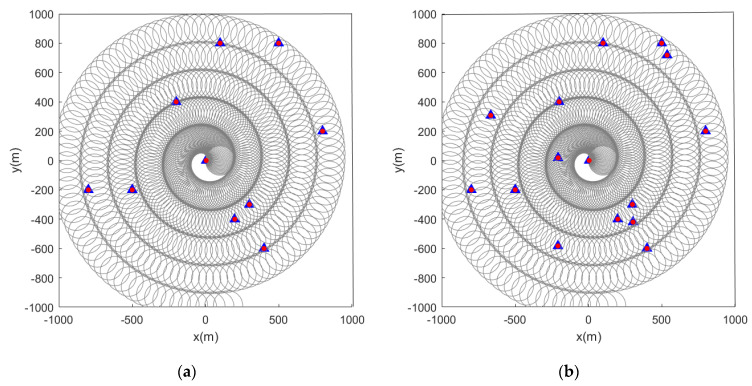
Estimated position of stationary feature points using the GLMB algorithm (the red and blue marks indicate the true and estimated positions, respectively, of the feature points in the scene). (**a**) Sensor detection radius is 100 m and there are 10 feature points in the scene; (**b**) sensor detection radius is 100 m and there are 15 feature points in the scene.

When the initial path of the sensor is a spiral curve, the feature point marking effect is as shown in the figure below. When the feature points in the scene are static, Figure 10 and Figure 11 are the simulation results obtained by using the LMB filtering method and the GLMB filtering method respectively.

**Figure 12:**
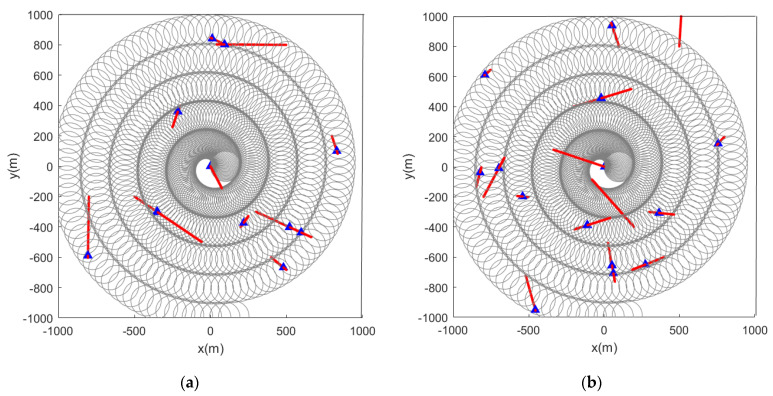
Estimated position of dynamic feature points using the LMB algorithm (the red track is the feature point motion track; the blue marks indicate the estimated positions of the feature points in the scene). (**a**) Sensor detection radius is 100 m and there are 10 feature points in the scene; (**b**) sensor detection radius is 100 m and there are 15 feature points in the scene.

**Figure 13:**
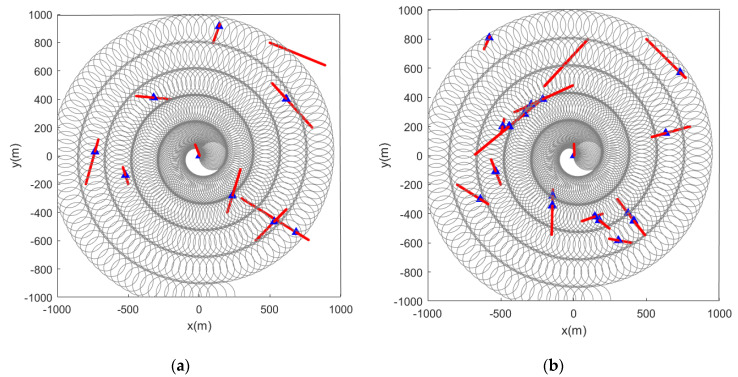
Estimated position of dynamic feature points using the GLMB algorithm (the red track is the feature point motion track; the blue marks indicate the estimated positions of the feature points in the scene). (**a**) Sensor detection radius is 100 m and there are 10 feature points in the scene; (**b**) sensor detection radius is 100 m and there are 15 feature points in the scene).

Subsequently, we change the feature points in the scene from static to dynamic. Figure 12 and Figure 13 show the situation where the feature points are detected and marked by the sensor during the movement.

From the pictures shown above, we can see that whether the feature points are static or dynamic, the red markers and blue markers have a high degree of coincidence. This shows that the LMB filtering method works well for feature point labeling. Furthermore, as the sensor travels along the initial path, its detection range covers almost the entire given area. This also shows that the initial path planning effect of the first stage is good.

**Figure 14:**
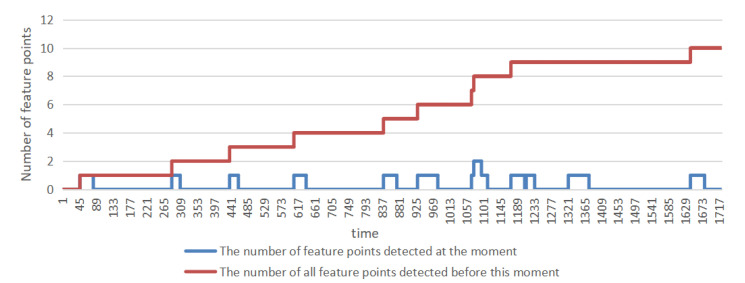
Estimation of the number of feature points. (The sensor route is a serpentine curve. The number of feature points is 10).

When there are ten feature points in the scene, Figure 14 shows the number of obstacles detected by the sensor at different times while walking along the path from the start point to the end point. This shows that the local path planning in the second stage has achieved the expected effect. The above simulation results show that the method proposed in this paper has a good effect on the estimation and detection of the position and quantity of feature points.

Consider a scene with ten feature points. The scenario ran for 100 time steps. T=1 is the sampling period. In the process of the sensor following the initial path, it is assumed that the given ten obstacles are independent of each other. The algorithm proposed in this paper can detect and mark multiple obstacles well. The simulation results also correctly estimated the location and number of feature points, and the incidence of false alarms and missed detections is very small. 

Then, we compared the performance of the two algorithms, LMB filtering and GLMB filtering. The 64-bit Win10 system PC CPU used to test the algorithm was: Intel(R) Core (TM) i5-10210U CPU @ 1.60 GHz 2.11 GHz. RAM: 12.0 GB. In the two cases where there are 10 feature points in the scene, we have performed ten simulation experiments in total. In each simulation experiment, the program runs for 100 time steps. For the above experimental simulation, the average CPU time is shown in the Table 2.

The results are further verified by the Optimal Sub Pattern Assignment (OSPA) distance. OSPA is a consistency measurement method for the overall performance evaluation of the feature point identification, detection, and tracking system. It defines a measurement distance in the system state space, which can be used to measure the error between the true value and the estimated value.

**Figure 15:**
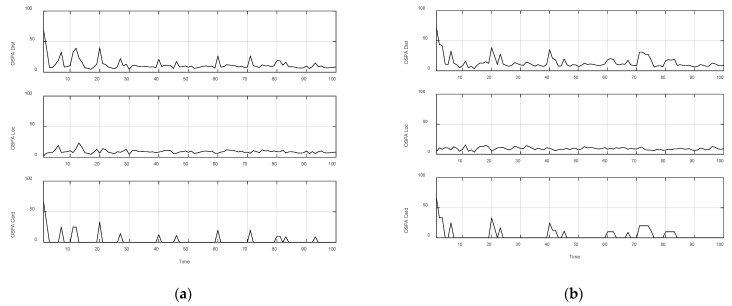
OSPA distance. (100 Mont Carlos: (**a**) OSPA distance of LMB filter algorithm; (**b**) OSPA distance of GLMB filter algorithm).

Define the true state set of the detected feature points as X and the estimated state set after the algorithm processing as Y. $m,n\in \left\{0,1,2,\ldots \right\}$, m≤n. x∈X, y∈Y represent the true state vector and estimated state vector of an independent feature point at a certain observation times respectively. The OSPA distance is defined as follows [34]:

(35) d¯(c)(X,Y)=1nminπ∈Πn∑i=1md(c)(xi,yπ(i))p+cp(n−m)1p

where $\prod_{n}$ represents taking all permutations and combinations of m elements from the sequence set {1,2,...}, and $\underset{\pi \in \Pi_{n}}{min}$ represents finding the pair with the smallest distance error from all the combination pairs of the estimated state set and the true state set. p=1 is the order, which can adjust the weight of OSPA for the distance error between the two sets. c=100 is a related parameter of the cut-off distance, which can adjust the correlation error weights of different numbers between the two sets. The OSPA distance is shown in Figure 15.

The experimental data in Table 2 show that the GLMB algorithm takes longer and is more computationally expensive than the LMB algorithm. It can be observed from the OSPA distance in Figure 15 that the LMB algorithm is slightly better than the GLMB algorithm in the detection and labeling effect of multiple feature points. The OSPA-loc curve shows that the two filtering algorithms have similar accuracy and can estimate the location of feature points more accurately, but the OSPA-card curve shows that the GLMB algorithm performs poorly in the estimation of the number of feature points. Considering the computational complexity of the algorithm, we believe that the LMB algorithm is more accurate for the detection and labeling of feature points, and the LMB algorithm can better estimate the state of multiple feature detection points under clutter conditions.

## 5. Conclusions

Aiming at the problem of low estimation accuracy of the location and quantity of map feature points in existing SLAM methods, this paper proposes a two-stage SLAM feature point detection and marking algorithm based on the LMB filter. We carry out path planning in stages and use measurement information to estimate the state of sensors and feature points. The B-spline curve is used to smooth the obstacle avoidance path of the sensor. Experimental simulation results show that the algorithm can effectively detect and mark feature points. However, when group feature points and extended feature points appear in the scene, further research is needed.

## Figures and Tables

**Table 1:** Sensor parameter setting.

Sensor	Parameter Settings
Straight-line speed	2 m/s
Arc travel speed	1 m/s
Detection step	10 m
Detection radius	50 m, 100 m, 150 m

**Table 2:** Comparison of the time spent in the experiments with the LMB and GLMB algorithms.

Algorithm	LMB	GLMB
Average time (100 MCs)	102.25	158.02
